# Physical, chemical and microbiological assessments of drinking water of small-layer farms

**DOI:** 10.4102/ojvr.v89i1.2067

**Published:** 2022-11-28

**Authors:** Eufrásia Augusto, Jescka Aleixo, Florentina D. Chilala, Abel G. Chilundo, Benígna Gaspar, Custódio G. Bila

**Affiliations:** 1 Paraclinic Department, Faculty of Veterinary Medicine, Eduardo Mondlane University, Maputo, Mozambique; 2 Clinic Department, Faculty of Veterinary Medicine, Eduardo Mondlane University, Maputo, Mozambique

**Keywords:** water quality, layers, total coliforms, *Escherichia coli*, risk factors, Mozambique

## Abstract

Water quality is critical for poultry farming. This study assessed the physical, chemical and microbiological quality of drinking water in small-layer farms in Southern Mozambique and identified potential risk factors for total coliform (TC) and *Escherichia coli* contamination of drinking water. In 20 farms, 57 samples were collected and examined for pH, nitrate content (NC), nitrite level (NL) and total hardness contents (TH). Furthermore, TC and *E. coli* growth were assessed at 37 °C. One hundred per cent of the drinking water was of acceptable quality in terms of pH (6.5–8.5), NC (50 mg/L) and NL (3 mg/L). Total hardness contents exceeded the recommended standard in 37.5% of borehole water samples and 91.7% of tap water samples, respectively. Total coliform and *E. coli* were found in 40% and 15% of water samples. Tap water samples had the greatest contamination, with TC and *E. coli* levels of 41.7% and 16.7%, respectively. Although not statistically significant, sampling from the beginning of the nipple line (*p* = 0.101, OR = 7.357, 95% confidence interval [CI]: 0.678–79.886) and not cleaning the rearing equipment regularly (*p* = 0.098, OR = 3.966, 95% CI: 0.766–20.280) were factors affecting the TC growth. Sampling from the tank water source (*p* = 0.001, OR = 0.005, 95% CI: 0.000–0.121) and borehole water source (OR = 13 585) and not cleaning the equipment consistently (*p* = 0.073, OR = 9.682, 95% CI: 0.810–115.68) were all factors affecting *E. coli* growth. It is concluded that the TH and microbiological quality of the drinking water of the study region are inadequate. Regular water quality assessments should be incorporated into Mozambican layer farm management to limit the potential for health concerns, and farmers should thoroughly clean and disinfect their rearing equipment.

**Contribution:**

We should incorporate regular water quality assessments into Mozambican layer farm management to limit the potential for health concerns, and farmers should thoroughly clean and disinfect their rearing equipment.

## Introduction

Water is required for the preservation of important activities, which is why living organisms must consume it to exist (Barros, Amaral & Rossi 2001). Water is essential to preserve homeostasis in living organisms, since it takes part in various processes in the body, including food digestion, nutrient absorption and transport, body temperature regulation and metabolite excretion (Lehninger, Nelson & Cox 1993).

Water is used for irrigation of fields, sanitary management, cleaning, disinfection of facilities and animal consumption in livestock production (Barbosa 2013; Simoni et al. 2015). Water may be obtained from a variety of sources, including springs, shallow wells, deep wells, lakes and streams (Amaral 2004). Because of open human defecation, inappropriate disposal of animal droppings and dead carcasses, pits built near water sources and manure dumping during the rainy season, surface water sources such as rivers and lakes are more prone to microbial contamination (Amaral 2004).

Water is essential in poultry farming for management, feeding, cleaning and environmental temperature regulation (Gama et al. 2008). Besides supplementation and disease treatment, water may be used as an electrolyte replacement therapy and a medication and vaccine delivery medium (Folorunso, Kayonde & Onibon 2014; Gama 2005).

Organic matter accumulation in water supply systems, such as reservoirs, drinkers and battery system pipes, may develop during layer bird rearing. Algae development, mineral deposition and dirt can result from accumulated organic matter, creating a suitable habitat for the multiplication of microorganisms in the water (Folorunso et al. 2014). The development of illnesses, reduced egg production and/or mortality of laying hens can all result from microbial contamination (Amaral 2004; Cardozo et al. 2015; Di Martino et al. 2018; El Allaoui, Rhazi Filali & Derouich 2016).

The use of water of poor physical, chemical or bacteriological quality can adversely affect livestock health and performance (Hapke 2000; Tabler 2003; Travel et al. 2007). When used for watering birds, the use of water of dubious quality negatively interferes with well-being and zootechnical indices and enhances the spread of diseases, causing serious economic losses (Tabler 2003).

Although water quality is a major factor in controlling animal health problems (Travel et al. 2006), the quality of drinking water used in the Mozambican poultry industry has never been evaluated. It was hypothesised that poor drinking water quality was contributing to Mozambique’s high morbidity and mortality rates. As a result, this study aims to evaluate the physical, chemical and microbiological water quality in small-layer farms in Southern Mozambique, as well as to identify potential risk factors for bacterial contamination of drinking water.

## Materials and methods

### Choice of farm sites

**FIGURE 1:**
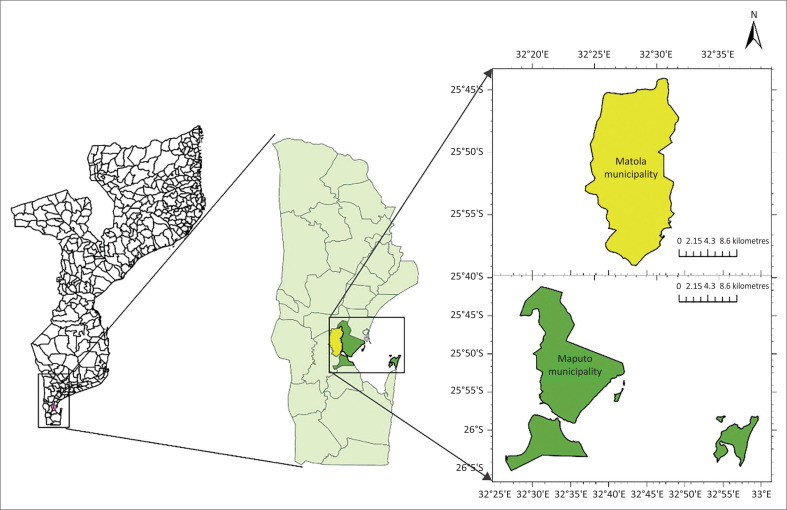
Study area, showing (a) Mozambique administrative division, (b) Maputo province and (c) Maputo and Matola municipalities.

The research was conducted in the municipalities of Maputo and Matola in Mozambique’s Southern Region. The municipalities of Maputo and Matola, respectively, have seven and 41 districts (Figure 1).

### Sampling

In October 2020, drinking water samples were collected once in each layer farm. Layer farms were picked based on convenience and nonprobabilistic sampling, and only immediately available layer farms were chosen, with a preference for small egg producers with less than 1000 layers. Using a battery rearing system, three samples were collected from each layer farm: (1) the tank water source, (2) the tank at the beginning of the nipple line and (3) the end of the nipple line. Only samples from the water supply and drinkers were taken from farms using the floor-rearing system. Water samples were taken in 500 mL sterile bottles using a specified process to avoid contamination (Di Martino et al. 2018; El Allaoui et al. 2016). They were then transferred to the laboratory at 4 °C and processed within 24 h for microbiological analyses and 48 h for chemical and physical analyses.

In 20-layer farms, 57 water samples were collected, with 17 using a battery cage rearing system and three using a floor rearing system, respectively.

### Physical and chemical analyses

A portable pH meter was used to determine the pH (Hach, England). The EDTA (ethylenediaminetetraacetic acid) titration method was used to determine the level of hardness (APAT 2003). Ion chromatography was used to determine the levels of nitrates and nitrites (APAT 2003).

### Microbiological analysis

#### Total coliforms

After suitable dilutions, 100 mL of water was filtered to be tested via a 0.45 µm pore size cellulose membrane filter to determine total coliforms (TC) (Standing Committee of Analysts 2002). Afterward, the membrane was put on lauryl sulphate agar (Oxoid, UK) and incubated for 24 h at 37 °C ± 0.5 °C. If colonies are yellow after primary culture in membrane lauryl sulphate agar (MLSA), water is most likely contaminated with TCs.

#### Faecal coliforms and *Escherichia coli* detection

The presence of faecal coliforms was detected in the same way as the TCs, with the exception that the incubation was carried out at 44 °C ± 0.5 °C for 24 h. A representative number of the usual colonies were subcultured on nutrient agar medium (Oxoid, United Kingdom [UK]) at 37 °C ± 1 °C for 24 h based on verified yellow colonies of faecal coliforms. To distinguish faecal coliforms, biochemical approaches were used. The presence of red colouring on the surface of the nutrient agar medium was then used to regulate indole synthesis by adding 0.2 mL – 0.3 mL Kovacs reagent (Merck Millipore, UK). All colonies exhibited a negative oxidase reaction, but a positive indole reaction turned out to be *Escherichia coli* in the end. If TCs or *E. coli* were found in a sample, it was contaminated.

### Questionnaire survey

Parallel to the collection of samples for physical, chemical and microbiological analyses, the layer farmers in this study completed a multiple-choice questionnaire validated by the authors of this paper that covered a variety of factors affecting water quality (including the number of birds in production, farmer experience with layer rearing, infrastructures, water sources, hygiene and equipment). The purpose of the questionnaire was to identify potential risk factors for bacterial contamination of drinking water in layers.

### Statistical analysis

For statistical analysis, all data were input into a Microsoft Excel^®^ spreadsheet (Microsoft Corporation, Redmond, Washington, United States) (MS Excel 2018) and exported to the Statistical Package for the Social Sciences (SPSS^®^) statistical software version 16 (IBM Corporation, Armonk, New York, United States) (IBM SPSS Statistics 16). For quantitative variables (pH, hardness, nitrates and nitrites), descriptive statistics were used, including the computation of mean, standard deviation, frequencies and percentages (water source, point of collection, washing facilities, washing equipment, hardness and pH limit).

A logistic regression model was used to investigate the risk factors associated with the quality of the water used to water laying hens, with the univariant analysis considering the response dichotomic and dependent variables (presence or absence of TCs and *E. coli*) and the study’s independent variables (water source, collection point, washing facilities, washing equipment, hardness and pH limit).

A multivariate analysis was performed on all variables that were significant in the univariate analysis (*p* < 0.05) to see if the existing association was because of confounding factors. The odds ratio (OR), which was obtained directly from the logistic regression estimates and provided in tables indicating the respective significance, was used to measure the degree of association between the independent and dependent variables.

Because there is no specific legislation for animal drinking, the physical, chemical and microbiological data from this study were compared with those from the European Council Directive 98/83/EC on the quality of water meant for human consumption (European Commission 1998).

### Ethical considerations

Ethical review and approval were granted by the Scientific Board of the Faculty of Veterinary Medicine, Eduardo Mondlane University, ethical clearance number: 891FAVET.

## Results

### Physical and chemical analysis

The physical, chemical and microbiological data are summarised in Table 1 and Table 2. The pH values are between 6.5 and 8.5. The concentrations of nitrate and nitrite were 50 mg/L and 3 mg/L, respectively. The values of the hardness parameters ranged from 50 mg/L to 740 mg/L.

**TABLE 1:** Chemical and physical analysis of drinking water in 20 farms at three sampling sites: the water source (A), the beginning (B) and the end of the nipple line (C). The data are expressed as median values with standard deviation.

Parameter	Minimum	Maximum	Sampling site					
			A		B		C	
			*n*	%	*n*	%	*n*	%
pH	6.22	7.5	6.85	0.28	6.82	0.26	6.68	0.25
Hardness (mg/L)	50	740	133.8	40.5	156.5	143.6	159.06	154.5
Nitrates (25 mg/L)	0.5	24.3	4.41	5.03	7.85	9.61	10.66	10.9
Nitrites (mg/L)	0.03	0.36	0.05	0.07	0.06	0.05	0.06	0.07

**TABLE 2:** Drinking water quality in layer farms supplied with either borehole or tap water in Matola and Maputo municipalities.

Water source: Parameter	Borehole				Tap water			
	Satisfactory quality		Unsatisfactory quality		Satisfactory quality		Unsatisfactory quality	
	*n*	%	*n*	%	*n*	%	*n*	%
pH	8	100.0	0	0.0	12	100.0	0	0.0
Hardness	8	100.0	0	0.0	11	91.7	1	8.3
Nitrates	8	100.0	0	0.0	12	100.0	0	0.0
Nitrites	8	100.0	0	0.0	12	100.0	0	0.0
Total coliforms	5	62.5	3	37.5	7	58.3	5	41.7
*E. coli*	7	87.5	1	12.5	10	83.3	2	16.7

### Microbiological analysis

Total coliform and *E. coli* contamination were found in 40% and 15% of the drinking water samples. Tap water samples had the greatest contamination, with *E. coli* and TC levels of 16.7% and 41.7%, respectively (Table 2).

### Potential risk factors for water contamination with total coliform and *Escherichia coli*

Table 3 shows the variables that were deemed risk factors for TC and *E. coli* in the water given to birds. The multivariate logistic regression model revealed no significant risk factors for the increase of TC in laying hens’ drinking water.

**TABLE 3:** Binary regression model of risk factors related to water quality for laying hens.

Variable	Risk factor	Category	OR	95% CI	*p*
Total coliforms	Sampling site	End of the nipple line	1	-	-
		Tank water source	0.250	0.050–1.248	0.091
		Beginning of the nipple line	7.357	0.678–79.886	0.101
	Water source	Tap water	1	-	-
		Borehole	1.238	0.227–6.751	0.805
	Equipment cleaning	Yes	1		-
		No	3.966	0.766–20.280	0.098
	Hardness limit	Unsatisfactory quality	1	-	-
		Satisfactory	0.451	0.074–2.750	0.388
	pH limit	Unsatisfactory quality	1	-	-
		Satisfactory	5.605	0.626–50.192	0.123
*E. coli*	Sampling site	End of the nipple line	1	-	-
		Tank water source	0.005	0.000–0.121	0.001
		Beginning of the nipple line	1.687	0.232–12.273	0.605
	Water source	Tap water	1	-	-
		Borehole	13.585	0.971–189.9	0.053
	Infrastructure cleaning	Yes	1	-	-
		No	0.038	0.000–15.298	0.285
	Equipment cleaning	Yes	1	-	-
		No	9.682	0.810–115.68	0.073
	Hardness limit	Unsatisfactory quality	1	-	-
		Satisfactory	0.003	0.000–0.110	0.002
	pH limit	Poor quality	1	-	-
		Satisfactory	18.192	0.814–406.3	0.067

OR, odds ratio; CI, confidence interval.

Although not statistically significant, the sampling point ‘beginning of the nipple line’ (*p* = 0.101, OR = 7.357, 95% CI: 0.678–79.886), the ‘satisfactory’ pH threshold (*p* = 0.123, OR = 5.605, 95% CI: 0.626–50.192) and the variable ‘no equipment cleaning’ (*p* = 0.098, OR = 3.966, 95% CI: 0.766–20.280) were linked to a higher risk of TC growth in the drinking water of laying hens.

When compared with the remaining locations, the probability of *E. coli* incidence or growth in drinking water for the layers relating to the collection point ‘tank water source’ (*p* = 0.001, OR = 0.005, 95% CI: 0.000–0.121) was statistically significant (beginning and end of nipple lines).

*E. coli* growth is higher in the variable ‘borehole water source’ (OR = 13.585). Consistent with this discovery, the variable ‘do not clean equipment’ also contributed to the increase in *E. coli* growth in laying hens’ water (*p* = 0.073, OR = 9.682, 95% CI: 0.810–115.68).

## Discussion

According to the authors’ knowledge, this is the first research to examine the physical, chemical and microbiological features of water used by small-layer farmers in Southern Mozambique, as well as the risk factors related to TC and *E. coli*.

In terms of the pH (6.5–8.5), nitrate content (NC) (50 mg/L) and nitrite content (3 mg/L), 100% of the drinking water given to the layers in the studied area was of satisfactory quality, while total hardness (TH) exceeded the recommended standard in 37.5% and 91.7% of water samples collected from boreholes and tap water, respectively. Using the pH parameter to evaluate the poultry water quality has sparked debate. According to Grizzle, Armbrust and Saxton (1996) and Cardozo et al. (2015), using water with a pH lower or higher than the recommended one (pH 4.8) did not affect poultry performance or water consumption. On the other hand, Carter and Sneed (1996) noticed a decline in broiler growth and feed conversion when they consumed water with a pH lower than 6. The impact of pH levels on water distribution systems in poultry farming management appears to be critical. Low-pH water (between 2 and 4) can cause corrosion in water transport equipment and limit the efficiency of detergents, disinfectants and vaccines (Gama 2005). As a result, knowing the pH of the water and, if required, making the appropriate changes is critical when providing these veterinary consumables (Gama et al. 2008).

High nitrate levels might indicate an overabundance of organic matter in the water supply because of the usage of animal manure or nitrogen-based fertilisers (Fonseca 2017). According to Gama et al. (2008), excessive amounts of nitrates in drinking water can induce poultry toxicosis by causing methaemoglobin to develop, which cannot transport oxygen to the cells. This toxicosis causes decreased growth and appetite in birds (Gama et al. 2008).

Because nitrates are a by-product of ammonium oxidation or nitrate reduction, their presence in water indicates recent pollution (Parron & Muniz 2011). Nitrite levels (NL) in poultry drinking water have been linked to decreased appetite, growth inhibition and lower laying rates in laying hens (Gama 2005).

Only one layer farm had an unacceptable hardness parameter from tap water supplied to this farm. Higher levels of hardness in drinking water can induce an unpleasant taste, reduced water consumption and decreased egg production in layer farming (Cardozo et al. 2015).

Also, increased levels of hardness promote calcium and magnesium deposition in the farm’s water transport tubes, resulting in precipitates that create an excellent environment for biofilm that contaminates the water (Cardozo 2012; Folorunso et al. 2014). Likewise, for the description of lower pH in drinking water, higher hardness negatively affects the efficiency of detergents, disinfectants, medicines and vaccines in poultry farm management (Di Martino et al. 2018).

Regardless of the water source, drinking water samples from 40% of the layer farms showed TC growth. When tap water is used, this number increases. The major issue related to TC contamination is a loss of birds’ immunity, which leaves them prone to opportunistic pathogenic infections that can harm poultry productivity and quality (Gama 2005).

*Escherichia coli* is a bacterium linked to colibacillosis epidemics in birds, which can lead to disease outbreaks. It is significant because it forms 95% of the bacteria that make up the faecal coliforms, the most well-known and studied group of bacteria. (Cardozo et al. 2015; Gama 2005). Poultry farms that used tap water from the public supply network had greater *E. coli* contamination (16.7%). This finding could be because the public water supply network’s piping system is very old and may have become contaminated with algae, dust and organic material, allowing bacteria to thrive (Folorunso et al. 2014).

Drinking borehole water was discovered to be a risk factor for the growth of TCs and *E. coli*. Borehole water, unlike tap water, is not chemically treated to prevent bacterial contamination. This lack of prevention may have favoured the higher contamination found in borehole drinking water.

In this study, water collection from point A (tank water source) was identified as a risk factor for *E. coli* growth in the layers’ drinking water. In Mozambique, small egg farm producers frequently ignore water reservoir hygiene because they are entirely unaware of the necessity for regular cleaning. This conclusion supports the findings of Folorunso et al. (2014), who said that water reservoirs can be a source of contamination even if the water is of high quality, especially when replenished without proper cleaning. Di Martino et al. (2018), on the other hand, observed a contrary outcome, with increased proliferation of *E. coli* and other microbes in the water collected at point C (nipple nozzle). Poor cleaning and disinfection, as well as exposure to birds, rodents and other animals, can all contribute to the contamination of water reservoirs (Folorunso et al. 2014).

This study concludes that the TH content and microbiological quality of the drinking water of the study region are inadequate. Therefore, to limit the risk of health problems, frequent water quality assessments should be incorporated into the administration of Mozambican layer farms. Farmers should also thoroughly clean and disinfect their farming equipment.
